# Commentary on Vulnerability and Resilience to Activity-Based Anorexia and the Role of Dopamine

**Published:** 2021-03

**Authors:** Jeff A. Beeler, Nesha S. Burghardt

**Affiliations:** 1 Department of Psychology, Queens College, CUNY, Flushing, NY, 11367 USA; 2 Department of Psychology, Hunter College, CUNY, New York, NY, 10065 USA; 3 Department of Psychology, The Graduate Center, CUNY, New York, NY, 10016 USA; 4 Department of Biology, The Graduate Center, CUNY, New York, NY, 10016 USA; 5 CUNY Neuroscience Collaborative, The Graduate Center, CUNY, New York, NY, 10016 USA

**Keywords:** Activity-based anorexia, Anorexia nervosa, Dopamine, Resilience, Vulnerability, Caloric restriction, Hyperactivity, Mice, Animal model, C57BL/6

## Abstract

Activity-based anorexia (ABA) is a commonly used rodent model of anorexia nervosa that is based on observations made in rats decades ago. In recently published work, we describe using this paradigm to model vulnerability and resilience to anorexia nervosa in mice, where vulnerability is characterized by hyperactivity and life-threatening weight loss and resilience is characterized by adaptation and weight stabilization. Using genetically modified hyperdopaminergic mice, we also demonstrate that increased dopamine augments vulnerability to ABA. Here, we briefly review our findings and discuss how obtaining vulnerable and resilient phenotypes enhances utility of the ABA model for understanding the neurobiological basis of anorexia nervosa. We comment on our dopamine findings and close by discussing implications for clinical treatment.

## Introduction

Anorexia nervosa (AN) is an eating disorder characterized by a fear of gaining weight and self-starvation, leading to life-threatening weight loss [1]. It occurs predominately in women and frequently begins during adolescence [2]. Although not part of the formal diagnostic criteria, exercise is an important aspect of this disorder, with up to 80% of AN patients engaging in excessive exercise [3-6]. Many patients report high levels of physical activity before the onset of the disorder, which they describe as progressively increasing during the most extensive periods of diet and weight loss [7]. While overactivity increases the risk of developing AN [7,8], including among athletes [9-13], most people diet and exercise without developing an eating disorder. Identifying factors mediating resilience and vulnerability to AN would contribute importantly to understanding the pathophysiology of the disorder.

We recently modeled vulnerability and resilience to AN in mice using the activity-based anorexia (ABA) paradigm [14]. ABA involves combining food restriction with unlimited access to a running wheel, making it ideal for studying how caloric restriction and increased physical activity interact to promote behaviors leading to weight loss. Early studies in rats have shown that these conditions lead to a paradoxical increase in wheel running, a self-imposed decrease in food intake, extreme weight loss and death, unless removed from the experiment [15]. We tested ABA in adult female C57BL/6N mice and observed that approximately half of the mice exhibited the expected increase in running and dramatic weight loss. These animals were all removed from the model when they lost at least 25% of their baseline bodyweight and were characterized as ‘vulnerable.’ The other half of the mice exhibited ‘resilience’ to ABA. Following an initial loss of bodyweight, resilient mice adapted to their limited food availability by reducing wheel running activity and progressively increasing their food intake, leading to weight stabilization. We also identified vulnerable and resilient phenotypes in our food restriction control mice (FR), which were food restricted for the same amount of time as the ABA group but were housed with a locked running wheel that prevented them from running. We then identified a role for dopamine in ABA vulnerability by testing dopamine hyperdopaminergic transporter knockdown mice. We found that increased dopamine promotes vulnerability by accelerating hyperactivity that occurs in response to caloric restriction. Here, we provide an expanded discussion of our study, highlighting novel aspects of our findings and implications for treatment.

## Enhancing the Utility of ABA as a Model of AN

The appeal of the ABA model is that it shares several features with AN, including increased physical activity, self-starvation, enhanced vulnerability during adolescence, and life threatening weight loss [16]. In contrast to the human disorder where food restriction is self-imposed, the experimenter gives animals in the ABA model a limited time (e.g. 1-2 hours) to eat as much as they want, usually at the onset of the dark cycle. Initially, food intake is low, which may not be purposeful, as animals do not yet know that food availability is restricted. However, with additional days of testing, we found that resilient mice housed with a freely turning (ABA) or locked running wheel (FR) steadily increase their intake, indicating that it is possible to consume sufficient food in the allotted time period (2 hours) [14]. The failure of vulnerable ABA and FR mice to do so despite the obvious need and the same duration of food access is consistent with a form of self-starvation.

The important role of food intake in ABA resilience was confirmed by the positive correlation between the amount of food resilient mice consumed each night and their change in bodyweight the next day. However, no such correlation was found in the vulnerable ABA mice and instead daily change in bodyweight correlated with wheel running. These results indicate that rapid weight loss in the vulnerable mice was driven by high levels of activity, which exacerbated the effects of caloric restriction. Upon closer examination of the distribution of wheel running, we found that daily weight change was associated with dark cycle running in all ABA mice, but only correlated with light cycle running in the vulnerable ABA group. Vulnerable mice also ran significantly more during the light cycle than the resilient mice or the wheel control mice (no food restriction), indicating a unique role for high levels of daytime activity in ABA vulnerability.

We conducted a more granular analysis of the characteristics of wheel running and found that *resilient* mice started running approximately 3 hours before food was available, consistent with food anticipatory activity (FAA) [17]. In contrast, vulnerable mice exhibited increased running throughout most of the light cycle, disrupting sleep patterns. While FAA reflects a shift in circadian activity entrained to food availability, presumably engaging mechanisms by which the availability of food modulates circadian cycling, the extreme light cycle running observed in the vulnerable mice appears to be more than entrainment. Instead it seems to reflect a failure of circadian cycling or a partial transition to diurnality, similar to what others have shown with food restriction [18-20]. Thus, we see that ABA evokes two distinct changes in running, one of which appears to be FAA and is associated with adaptation and resilience, and one that reflects disrupted circadian cycling and is associated with vulnerability. In terms of their clinical relevance, to our knowledge there are no reports of FAA-like behavior or symptomology in AN, but there are reports of disturbed sleep patterns in AN [21], adding to the validity of our model of AN vulnerability.

Since the early descriptions of the ABA model by Routtenberg and Kuznesof in 1967 [15], it has been known that food restriction can cause rodents to run themselves to death. A central question has been why animals that have access to food (albeit limited) would expend so much energy instead of preserving energy until food arrives. The life-threatening light cycle running found in our vulnerable mice likely reflects the engagement of ‘crisis foraging,’ in which a state of starvation upregulates drive for activity to increase the probability of finding food. Under conditions of net negative energy balance, where the animal will die without additional food, it makes sense to drive energy expenditure in a final effort to find food. Maladaptive activation of the neural circuits underlying foraging may account for the excessive running exhibited by vulnerable mice. However, this does not explain why vulnerable mice, like those with AN, fail to eat sufficient amounts of food when it is available. It has been proposed in the Adapted to Flee Famine Hypothesis that hyperactivity and self-imposed food restriction were once evolutionarily adaptive responses in ancestral nomadic foragers [22]. According to the hypothesis, the most essential action to ensure survival during local famine is to flee to a new environment containing food. In doing so, the individual needs to increase physical activity and direct attention away from searching for food in the current environment, which requires ignoring food and denying starvation. Weight loss has been suggested to trigger these archaic adaptions in genetically susceptible individuals [22], an idea that might also be true for the vulnerable mice in our model.

Our detailed analysis of wheel running revealed that in addition to running more throughout the light cycle, vulnerable mice exhibited a dramatic burst in light cycle running shortly before requiring removal from the experiment. This sudden increase was quite dramatic, with mice running an *additional* 11,000-56,000 revolutions in one light cycle, which is a 3.0-15.6 kilometer *increase* in running from the previous light cycle. We detected the same phenomenon in younger animals and hyperdopaminergic animals, both of which are highly vulnerable to ABA. This has not been reported previously, so there is little upon which to base an interpretation of this phenomenon. However, the abrupt and dramatic nature of this increase together with its consistent occurrence prior to reaching a dangerously low bodyweight (75% of baseline weight), and its absence in resilient mice, is suggestive of some equally abrupt change in underlying pathophysiology or neuroadaptation. Progression from normal bodyweight to life threateningly low bodyweight is compressed in the ABA model to a few days. In AN, a presumably similar sequence of analogous neural and physiological adaptations occurs over the course of months and years. While it is difficult to speculate whether this abrupt increase in activity reflects any corresponding phenomenon in human AN, it is worth noting that individuals with AN have described exercising the most during the most extreme periods of diet and weight loss [7]. Moreover, in qualitative, narrative studies, one theme that emerges in patients’ stories is the rapidity at which an exercise regimen becomes compulsive [23]. The abrupt, dramatic increase in light cycle running we observe in vulnerable mice may be comparable to how AN patients transition from experiencing exercise as voluntary to experiencing it as compulsive.

The importance of excessive wheel running in achieving life-threatening weight loss in the ABA model was demonstrated in early studies conducted by Routtenberg and Kuznesof [15]. In contrast to food restricted rats housed with a running wheel, they found that those without a running wheel (FR control group) maintain a stable bodyweight. This necessary role of exercise in ABA is actually implicated in the name “activity-based anorexia.” Some subsequent studies have replicated this finding in the FR control group [24,25], while others have not [26,27], results that are likely attributable to methodological differences across experiments (see Supplemental Discussion of [14] for details). In our study, we found that some mice in the FR control group do exhibit vulnerability, indicating that the running wheel may not be essential under all conditions and that the *primary* driver of the vulnerable phenotype is caloric restriction, not exercise. This is not to minimize the importance of wheel running, which we show facilitates weight loss in vulnerable animals. Given that not all AN patients engage in excessive exercise, this FR control group provides an opportunity to model vulnerability and resilience to dieting alone. Studying both the ABA and FR groups allows us to observe the full spectrum of behavioral dysregulation of energy balance, where hyperactivity is one part of the equation.

In sum, we provide a mouse model of vulnerability and resilience to AN in which contrasting behavioral adaptations to both food intake and activity lead to distinct outcomes (i.e. dangerous weight loss vs. weight stabilization). Regina Casper has called AN a ‘disorder of energy homeostasis,’ [28,29] which aptly captures the derangement of both activity and consumption we observe in our vulnerable ABA mice. Bulik and colleagues [30,31] have characterized AN as both a metabolic and psychiatric disorder in their genetic work. In terms of treatment, the psychological aspects of the disorder are often the primary focus with the expectation that improvements in behaviors mediating the metabolic aspects will follow. Like any animal model, we are limited in our ability to capture many psychological aspects of this complex disorder. However, we concur with Södersten and colleagues that the neurobiological and endocrine adaptations that underlie AN are the root cause of the disorder and likely drive psychiatric symptoms [32-34]. The extent to which our vulnerable phenotype exhibits a pattern strikingly similar to AN suggests to us that the adaptations that underlie vulnerability to ABA and FR reflect similar pathophysiological adaptations to caloric restriction that underlie AN. It is hoped that future studies focused on identification of these adaptations will provide much needed insight into the neurobiological basis of AN.

## Dopamine in AN

AN has long been associated with abnormalities in the dopamine system [35-37]. A persistent question in the field is whether these changes reflect preexisting individual differences in dopamine or arise secondary to the disorder, i.e., trait or state [38-40]. While our data with hyperdopaminergic mice indicate that preexisting differences in dopamine can modulate vulnerability to ABA, i.e., be a trait risk factor, this does not rule out the possibility that the dopamine system also changes over the course of the disorder. As many have hypothesized, these secondary changes could contribute to the development and maintenance of the disorder [40-46]. For example, in some individuals caloric restriction and exercise could induce an upregulation in the dopamine system that in turn facilitates the development of the disorder. An increase in dopamine function prior to diet and exercise, such as that found in our hyperdopaminergic mice, could predispose individuals to these dopaminergic neuroadaptations, thereby increasing AN risk. However, direct evidence in humans for preexisting alterations in dopamine function, such as prospective studies or genome-wide association studies, is currently lacking.

Interestingly, we found that higher baseline levels of dopamine did not affect how mice responded to food restriction in the absence of a running wheel. That is, hyperdopaminergic mice and wild-type littermates ate the same amount of food when tested in the FR control conditions. Similarly, both groups ran the same amount when housed with a running wheel and given unlimited access to food (wheel control conditions). Therefore, preexisting increases in dopamine did not simply increase vulnerability to food restriction or promote hyperactivity. Instead, enhanced dopamine interacted with the *combination* of wheel running and food restriction to promote vulnerability to ABA. These results support the idea that there are neuroadaptive changes arising from the combination of diet and exercise that could interact with preexisting alterations in dopamine to facilitate the development and maintenance of AN. Such dopamine-mediated increases in AN risk may not result when dieting occurs in the absence of exercise or vice versa.

In contrast to our finding that hyperdopaminergic mice are more vulnerable to ABA, Foldi et al. [24] demonstrated that pharmacogenetic activation of dopamine in the mesolimbic pathway via virally expressed DREADDs (Designer Receptors Exclusively Activated by Designer Drugs) rescues rats from ABA. In that study, DREADD expression was not restricted to dopamine cells, leaving open the possibility that activation of midbrain GABAergic cells contributed to the rescue. Moreover, the use of systemic CNO to drive DREADD activation and the subsequent conversion of CNO to the antipsychotic clozapine [47] further complicates the interpretation of those findings. Clozapine has established effects on weight gain [48] and may have recruited additional neurotransmitter systems in that study [49]. Contradictory evidence about the direction of putative dopamine alterations in AN has been a persistent theme for decades [36,37]. While our data with hyperdopaminergic mice suggest that preexisting alterations in the dopamine system could enhance vulnerability to the disorder, it remains unknown how caloric restriction in combination with exercise might alter dopamine to promote the development of AN.

Diet and exercise affect many endocrine and neural substrates, several of which modulate dopamine [40]. These include, but are not limited to, insulin, leptin, ghrelin, glucocorticoids, orexin, endogenous opioids, and estrogen. If resulting changes in dopamine function do contribute to the development of AN, then preexisting genetic differences in any of these systems could affect AN risk. In this way, the risk for a single central pathophysiological change, alterations in dopamine, may be polygenic. This also opens up the possibility that preexisting alterations in dopamine function may not be required for an individual to be vulnerable to AN, even if changes in dopamine function crucially contribute to the development of the disorder. For example, we report in our study that adolescent female mice, which are highly vulnerable to ABA, have normal levels of dopamine at baseline and normal expression of dopamine-associated proteins in the striatum. Perhaps adolescent mice are more sensitive to food restriction and wheel running, leading to higher activation of one (or more) of the factors listed above that modulate dopamine, thereby triggering maladaptive changes in this neurotransmitter system and excessive weight loss. Future studies tracking endocrine and dopamine changes across the course of ABA are required to test this possibility.

Discussions of dopamine in AN primarily refer to dopamine in the ventral tegmental area, a subregion of the midbrain with projections to the prefrontal cortex and limbic regions (e.g., nucleus accumbens, hippocampus and amygdala). However, our genetic knockdown of the dopamine transporter was not restricted to the midbrain and increased dopamine in other regions may have contributed to our findings. One candidate region is the hypothalamus, where dopamine may play a crucial role in energy homeostasis [50-52]. The hypothalamus and midbrain are reciprocally connected regions that modulate each other [53,54], and together could orchestrate the adaptive and maladaptive behaviors associated with the resilient and vulnerable phenotypes we describe.

## Implications for Treatment

An unusual finding in our study was that among the resilient mice, those that exercised showed better adaptation to food restriction than those that did not. Specifically, resilient mice in both the ABA (freely turning wheel) and FR (locked wheel) conditions increased their consumption and stabilized bodyweight, but the resilient ABA mice consumed more food than the resilient FR mice and they maintained their bodyweight at approximately 90% of baseline, despite increased energy expenditure. In contrast, the FR mice maintained their bodyweight at about 80-85% of baseline. In the same way that voluntary wheel running appears to augment and accelerate failure to adapt to food restriction in vulnerable ABA mice, it seems to equally augment and accelerate adaptation among resilient ABA mice. Clinically, conventional approaches to treatment often prevent exercise activity in patients to prevent further weight loss and promote weight gain [55,56]. In severely malnourished states, exercise can also increase the risk of bone fractures, cardiac incidents and other potential injuries [57]. However, in recent years clinical studies have begun challenging this approach by investigating the therapeutic effects of moderate exercise. Accumulating evidence indicates that ‘appropriate’ exercise may actually be beneficial and improve treatment outcomes [58-61], results that are consistent with our findings in resilient mice.

Given the compulsive nature of exercise in AN, developing a regimen of ‘appropriate’ exercise within a treatment plan may be difficult to implement. That said, Södersten and colleagues have long argued that restoring caloric intake is the single most critical aspect of treatment [33]. They suggest that restoring bodyweight allows deranged neuro- and endocrine adaptations to normalize, facilitating psychological and behavioral changes that support recovery. Therefore, it may be that a more effective treatment plan would be to restore caloric intake and then after some weight restoration, allow for moderate exercise activity. This would be analogous to shifting patient behavior so they no longer exhibit the vulnerable phenotype (low food intake, high levels of exercise) and instead exhibit the resilient phenotype (progressive increases in food intake, moderate levels of exercise). In this clinically induced shift, the positive role of exercise in treatment and recovery may be as significant as the negative role it plays in the pathophysiology of the disorder.

The development of an effective pharmacological treatment for AN continues to be elusive. Our finding that increased dopamine enhances vulnerability to ABA supports dopamine as a potential therapeutic target, an idea that has persisted since the 1970s [35]. However, dopamine acting antipsychotic drugs have shown little to no efficacy in AN [62-66]. Our poor understanding of *how* dopamine is altered and its exact role in AN makes it difficult to ascertain why antipsychotic drugs have not been effective. Directly targeting D2 dopamine receptors with antipsychotic medications may be too simplistic an approach, as it assumes a direct link between AN behaviors and receptor activation. Several theories have suggested that increased dopamine drives behaviors that mediate AN through effects on reinforcement learning and brain plasticity rather than directly causing self-starvation or hyperactivity [41,44,67]. Such a complex role of dopamine highlights the importance of uncovering exactly how it changes over the course of the disorder. A more nuanced, physiological approach may be to target other systems that interact with and modulate dopamine, such as insulin, leptin, ghrelin, glucocorticoids, orexin, endogenous opioids, and estrogen. If the interaction of these potential targets with the dopamine system during physiological adaptation to caloric restriction and elevated exercise drives changes in dopamine that consequently promote maladaptive behaviors underlying AN (e.g., [43]), then indirectly targeting dopamine may be a more effective strategy.

Overall, a combination of diet and exercise appears to create fertile ground for the development of AN, and yet only a relatively small subset of individuals will actually develop the disorder. While much of the literature on AN risk has focused on psychological factors, the ABA model strongly suggests a substantial biological component. Indeed, AN has high heritability estimated at 50-60% [68,69]. Our study increases the utility of the ABA model by adjusting the parameters such that it yields both vulnerable and resilient mice, where resilience is defined by adaptation, weight stabilization and survival and not by delayed life-threatening weight loss (e.g., [27,70,71]. With clearly distinguishable phenotypes, the model can facilitate better understanding of genetic risk factors and the neural and endocrine adaptations that underlie the development of AN, potentially opening new avenues for treatment and prevention of the disorder.
